# Energy-Efficient Algorithms for Path Coverage in Sensor Networks

**DOI:** 10.3390/s23115026

**Published:** 2023-05-24

**Authors:** Zhixiong Liu, Wei Zhou

**Affiliations:** 1 School of Computer Science and Engineering, Changsha University, Changsha 410022, China; 2 Department of Computer Science and Software Engineering, Swinburne University of Technology, Hawthorn 3122, Australia; weizhou@swin.edu.au

**Keywords:** sensor network, path coverage, least movement, curve disjunction, weighted bipartite matching

## Abstract

Path coverage attracts many interests in some scenarios, such as object tracing in sensor networks. However, the problem of how to conserve the constrained energy of sensors is rarely considered in existing research. This paper studies two problems in the energy conservation of sensor networks that have not been addressed before. The first problem is called the least movement of nodes on path coverage. It first proves the problem as NP-hard, and then uses curve disjunction to separate each path into some discrete points, and ultimately moves nodes to new positions under some heuristic regulations. The utilized curve disjunction technique makes the proposed mechanism unrestricted by the linear path. The second problem is called the largest lifetime on path coverage. It first separates all nodes into independent partitions by utilizing the method of largest weighted bipartite matching, and then schedules these partitions to cover all paths in the network by turns. We eventually analyze the energy cost of the two proposed mechanisms, and evaluate the effects of some parameters on performance through extensive experiments, respectively.

## 1. Introduction

Wireless sensor networks (WSNs) are generally regarded as a type of smart network which consists of multiple nodes with limited capabilities on energy, computation, and storage [1]. In certain popular scenarios of these networks (e.g., target tracing), people have a great interest in guarding an object’s moving trail. This type of problem is called path coverage [2,3]. For nodes that are normally deployed at some sparse regions at random, a portion of them need to be moved to cover the path of the target, and minimizing the total moving distance of nodes should be considered seriously in resource-constrained networks [4]. Moreover, it is usually feasible to schedule these intensively deployed nodes for monitoring in some efficient ways to extend the life cycle of the network [5].

On minimizing the movements of nodes in sensor networks, some solutions have been presented for target coverage rather than for path coverage. Considering this, we propose a heuristic algorithm for minimizing the movements of nodes in sensor networks for path coverage for the first time in the heuristic algorithm; each path is divided into some discrete points by utilizing the curve disjunction technique. After finding out all redundant sensors and paths, sensors are then moved gradually to cover the given path under a set of regulations. As a result, path coverage can be achieved with the fewest movements of sensors.

On maximizing the lifetime of sensor networks, plenty of algorithms have been proposed, but most of them mainly focus on scenarios of point coverage and region coverage, rather than on those of path coverage. Therefore, we propose another heuristic algorithm on path coverage with the largest monitoring lifetime, which suits common sensor networks. Nodes are first divided into groups that can cover the path independently in the algorithm. Then, the largest weighted bipartite matching is utilized to schedule the nodes in each group. As a result, maximizing the lifetime of the network can be achieved.

The main contributions of this paper can be summarized as follows:

First, the problem of path coverage with the fewest movements of sensors is proved to be NP-hard, and then a new algorithm is proposed. The adopted curve disjunction technique overcomes the limitations of the linear path, which is a challenge in most of the existing schemes. Additionally, the effects of some parameters on moving distance are evaluated through further simulations.

Second, we present a heuristic algorithm for the problem of maximizing the lifetime of sensor networks on path coverage. In the experiments, we inspect not only the relationship between the number of nodes and lifetime of coverage, but also the relationship between the initial battery level and lifetime of coverage.

The rest of the paper is organized as follows. Related works on coverage in sensor networks are surveyed in Section 2. The algorithm on path coverage with the fewest movements in sensor networks is presented In Section 3, followed by Section 4, which presents the details of the largest lifetime of path coverage. Finally, Section 5 concludes the whole work.

## 2. Related Work

In recent years, some works have been presented to cope with the moving problems of sensors [5,6,7,8,9]. Liao et al. [5] gave a solution for the Mobile Nodes Deploying problem (MND) by decomposing it into two sub-steps. The first is the Target Coverage problem (TCP), and the second is the Network Connectivity problem (NCP). The named TCP problem concerns, supposing there exist *m* objects and *n* randomly located nodes, moving nodes to cover all objects with the least distance. It proved the NP-hardness of TCP, then solved TCP and NCP one by one, and finally addressed the MND problem through the combination of their solutions. Hefeeda et al. [6] proposed an approximated algorithm for *k*-coverage in intensively deployed sensor networks. Without considering the mobility of sensors, Zhang et al. [7] proposed a probabilistic mechanism. However, due to the fact that each node only works for another one that is out of service, the largest moving distance of each node is restricted. Attea et al. [8] modeled the Minimum Set Covering Problem (MSCP) to move sensors that can achieve energy-efficient coverage. Han et al. [9] proved that finding the largest number of crossed barriers is NP-hard, and presented a heuristic algorithm called MSPA based on a multi-round shortest path to solve the problem.

For maximizing the lifetime of sensor networks, many algorithms have been presented in recent years [7,10,11,12,13,14,15,16,17,18]. Dhawan et al. [10] proposed a mechanism to prolong the lifetime of the network by constructing the Lifetime dependency Graph (LG) to select a subset of sensors that can cover the target. Mini et al. [11] utilized the artificial bee colony algorithm and particle swarm optimization to schedule sensors to achieve the theoretical upper bound of a network’s lifetime. Abrams et al. [12] proved the problem of maximizing sensor network lifetime to be NP-Complete and presented a heuristic mechanism to solve it. Based on the method of separating nodes into the largest quantity of disjoint collections, Cardei et al. [13] proposed a scheme to extend networks’ monitoring lifetime. In the case of bounding the density of targets, Lu et al. [14] presented a PTAS mechanism to prolong the monitoring lifetime of the network and verified that, even in specialized conditions for nodes possessing the same sensing range and transmission range, the problem of scheduling nodes aimed to maximized lifetime belongs to NP-hard. Pointing to the scenario where nodes form a barrier to trace moving objects, Zhang et al. [7] provided an approximated algorithm for path coverage without considering energy optimization. Gu et al. [15] had a joint consideration of energy efficient routing and sleep scheduling, and mathematically formulated the lifetime maximization problem under multiple constraints (i.e., routing, end-to-end delay bounds, sleep scheduling, the energy consumption of transmission, receiving and listening, etc.). Considering that the formulated problem is a mixed integer non-linear programming (MINLP) problem and NP-hard to solve, they relaxed it into a linear programming (LP) problem and solved the relaxed problem for the upper bound. Weng et al. [16] proposed an Efficient *k*-Barrier Construction Mechanism (EBCM), aiming to schedule the sleep-wake time of all the constructed barriers to achieve energy balance. Yoon et al. [17] derived the upper and lower bounds on the coverage of a 2-D deployment of static sensors, and then used these bounds in constructing a method of estimating the coverage of deployment by assuming that there are only pair-wise intersections between the disks representing the range of each sensor. Ma et al. [18] proposed a hybrid strategy-improved butterfly optimization algorithm based on the elite-fusion and elite-oriented local mutation strategies.

As can be seen from the above, most of the existing works focus on point coverage or region coverage-related problems, while little attention has been paid to path coverage. This paper utilizes curve disjunction and largest weighted bipartite matching to achieve energy-efficient path coverage in sensor networks. The comparison of the main coverage algorithms is illustrated in Table 1.

## 3. Least Movement of Sensors on Path Coverage

### 3.1. Problem Description

We first define the problem under study and then prove its hardness in this section.

**Definition** **1.** *Path coverage with least nodes’ movements Problem (noted as PCP). Supposing there exists a path P and a group of nodes belonging to collection S, move nodes to cover P with the least distance making the probability of each point in P being covered not less than d. The covering probability of a point is defined as follows in [3].*

**Definition** **2.** *Covering probability. The probability of node s covering object t is*

(1) $$P_{ts}=\left\{\begin{array}{l} 1,\;\;\;\;\;\;\;\;0\leq |s,t|\leq R_{1};\\ e^{\beta (dis(s,t)-R_{1})},\;R_{1}\leq |s,t|\leq R_{2};\\ 0,\;\;\;\;\;\;\;\;|s,t|\geq R_{2}. \end{array}\right.$$

*When point j is covered by several nodes (s*_1_, *s*_2_, …, *s_N_), then P_j_ is the accumulative covering probability of s*_1_, *s*_2_, …, and *s_N_.*

(2) $$P_{j}=1-\prod_{i=1}^{N}\left(1-P_{ji}\right)$$

*Note S(v) and P(u) as the collection of nodes covering point v, and the collection of points being covered by node u, respectively. Supposing Q is the collection of multiple disconnected points (p*_1_, *p*_2_, …, *p_N_), we note M_j_(p_i_) as p_i+j_ and N_j_(p_i_) as p_i−j_, respectively.*

**Theorem** **1.** *PCP is an NP-hard problem.*

**Proof.**  The proof is a deduction of TCP [5], which has already been verified as an NP-hard problem. First, supposing there exist *m* objects and *n* nodes in TCP, move nodes to cover all objects, making the total movement the least. Second, construct PCP as follows: sort all objects according to the x-axis, then connect all objects successively to form a path *P*, *and* finally, move nodes to cover *P* with the least distance.

In one case, suppose *m* new locations of nodes covering all objects with the fewest movements existed. The *m* discrete points mentioned above in *P* can be covered for the reason that *P* is constructed by *m* objects. As a result, *m* nodes in the new locations can cover *P* with the fewest movements in PCP.

In another case, assuming *m* new locations of nodes existed, which led to covering *P* with the fewest movements. It is easy to know that there are *m* discrete points in *P* being covered by these nodes. Here, *m* points are corresponding objects in TCP; thus, these new locations of nodes can cover all objects with the fewest movements in TCP.

Therefore, PCP is also NP-hard. □

### 3.2. The Least Movement Algorithm

The algorithm to solve PCP is divided into three steps: (1) separate the path into a partition of discrete points; (2) for nodes that do not cover any point in the path, move each of them to cover the closest point in *P*; and (3) move nodes to cover all discrete points with the least distance.

#### 3.2.1. Path Disjunction

Path disjunction is to separate the given path *P* into multiple points in collection *Q*, which is implemented as follows: define a step-size threshold *d*, and in each run, fetch a point in *P* whose range is *d* far away from the former one according to the x-axis; carry this out continuously, until all of the *n* points are included in the collection *Q* finally. The corresponding pseudo-code is shown in Algorithm 1, which consumes time O(*n*).
**Algorithm 1:** Curve disjunction1. Let step-size *d* be (big-coordinate − min-coordinate)/*n*; 2. Let the collection *Q* be NULL; 3. For *i* from 1 to *n* do 4. *x_i_* = (*i* − 1/2)*d*; 5. Insert the fetched point (h(*x_i_*), *x_i_*) into collection *Q*; 6. Return collection *Q*.

#### 3.2.2. Initial Movement

After deployment, we need to find out the nodes which are free of work and move each of them to cover at least one point, respectively. The aim of the initialized movement is illustrated below. Preset a group of nodes belonging to collection *S* and a group of points belonging to collection *Q* in path *P*; move each node that is not covering any point in *P* to cover the closest point in *P*. Here, we note the distance between the corresponding node and the point as *R*_2_. The pseudo-code is shown in Algorithm 2. As finding out the closest point in the path runs in O(*n*), judging every node needs time O(*nm*).
**Algorithm 2:** Initializing movement/* Input: points in collection *Q* with size *n* in path *P*, deployed node collection *S* with size *m*. Output: move *S* shortest such that for *s_i_* in *S*, there exists *p_j_* in *Q*, where |*s_i_*, *p_j_*| ≤ *R*_2_; */ 1. For *i* from 1 to *m* do 2.    If |*s_i_*, every point in *Q*| > *R*_2_, then 3.    move *s_i_* to its closest point *p_j_* such that |*s_i_*, *p_j_*| = *R*_2_; 4. Return.

#### 3.2.3. Last Movement

After the initial movement, we need to move nodes further such that all discrete points in the path are covered. We first define the redundant node and redundant path, respectively, and then present the moving regulations.

**Definition** **3.** *Redundant node. Assume that the covering probability of point j is not smaller than d in Q, i.e., P_j_ ≥ d; if the equation still works after taking node s_i_ out of S(j), then node s_i_ is called a redundant node.*

In order to find out a redundant node of *p_i_*, we need to judge whether there is a redundant node: if point *j* has an initial covering probability not less than *d*, which becomes less than *d* after removing node *s*, then *s* is not redundant. Further, we need to check if the redundant node is closest among all neighbors of *i*. The pseudo-code of finding a redundant node is listed in Algorithm 3, which runs in time O(*nm*^2^).
**Algorithm 3:** Find-redundant-nodes/* Input: points collection *Q* with size *n* in *P*, nodes collection *S* with size *m*, point *i*. Output: the closest redundant node in S(*i*) to *N*(pos(*i*)).*/ 1. For (1≤ *i* ≤ *n*) & (*P_i_* ≥ *d*), then 2.       If *P_i_* < *d* after moving *s*, then 3.           Return Ø; 4. If *orient* = left, then 5.       Set *N*(pos(*i*)) as *M_j_*(*p_i_*) 6. Else if *orient* = right, then 7.       Set *N*(pos(*i*)) as *N_j_*(*p_i_*) 8. Else if *orient* = self 9.       Set *N*(pos(*i*)) as *i*; 10. Take *i* out of *Q*; 11. If exist a closest redundant node *s* to *N*(pos(*i*)), then 12.         Insert *i* into *Q*; 13. Return *s.*

**Definition** **4.** *Redundant path. For points p*_1_*, …, p_i_, assume the covering probability of p*_1_* is less than d, and there are no redundant nodes in p*_2_*, p*_3_*, …, and p_i−*1*_, except that p_i_ possesses a redundant node s_i_. Find a partition of nodes s*_2_*, …, s_i_, and move s_i_ to monitor p_i−*1*_, …; similarly, move s*_2_* to monitor p*_1_*, such that all points have covering probability not smaller than d. Then, the path (p*_1_*, p_i_) is redundant.*

We then present the heuristic regulations to move nodes.

**Regulation** **1.** *To a point p_j_, whose covering probability is smaller than d, examine all redundant nodes in collection S(p_j_) and move the closest one to guarantee the probability of covering p_j_ is not smaller than d.*

**Regulation** **2.** *Given path (p_i_, p_k_) and a redundant node in S(p_j_), if j = i − *1*, we move the closest redundant node to guarantee p_j_ is not smaller than d; otherwise, we move the closest redundant node of p_j−*1*_ to guarantee p_j−*1*_ not smaller than d.*

When *p_j_* is smaller than *d*, we chase a redundant node in S(*p_j_*). If it works, Regulation 1 is applied to move nodes. Otherwise, we have to chase a redundant path. If it works, Regulation 2 is used.

The total algorithm for solving PCP is given in Algorithm 4. Combined with curve disjunction, initial movement, and last movement, PCP can be solved in O (*n*^4^*m* + *n*^3^*m*^2^).
**Algorithm 4**: PCP solution/* Input: points collection *Q* with size *n* in *P*, nodes collection *S* with size *m.* Output: move *S* shortest distance to cover *P*, or report failure.*/ 1. Rank all points in line with x-axis; 2. Compute covering probability of all elements in collection *Q*; 3. Let *mv_len* = 0; 4. Initializing node movement; /*Algorithm 2*/ 5. For (0 ≤ *i* ≤ *n*) & For (1 ≤ *j* ≤ *n*) 6.       If exists redundant node *s* in S(*M_i_*(*p_j_*)) or S(*N_i_*(*p_j_*)), then 7.         When (*i* = 0), move *s* to *s_0_* according to Regulation 1; 8*.         mv_len* = *mv_len* + |*s_0_*, *s*|; else 9.       If *s* in S(*M_i_*(*p_j_*)), then let *orient* be left 10.       Else let *orient* be right. 11.        If *x*_0_ in *M_i_*(*p_j_*) or *N_i_*(*p_j_*) possesses a redundant node; 12.           Note path (*p_f_*, *p_f−_*_1_, …, *p*_1_); 13. For (*r* = *f*, *r--, r* ≥ 1) 14.        If exists redundant node *s* in S(*p_r_*), then 15.           When (*r* ≥ 2) then move node according to Regulation 2. 16.        Else move *s* according to Regulation 1; 17. Move node to *s*_0_; add |*s*_0_, *s*| to *mv_len*.

#### 3.2.4. Simulations

To confirm the effectiveness of the proposed scheme, we establish the simulated platform using the Python and C++ languages. Due to the limitation of space, we only present results for the changing conditions of the total moving distance of nodes, according to variations of the number of discrete points in the path, parameters *R*_1_ and *R*_2_, respectively. The size of the monitoring region is 100 × 100 m^2^, where 120 nodes are located at random in it. Additionally, we use function *y* = 0.1 × (*x* − 10) × (*x* − 20) (0 < *x* < 100) to generate points in the path. The threshold of covering probability *d* is set as 0.5, and *β* is set as 0.5. The sensing radius *R*_1_ changes from 0 to 2.5 m, and *R*_2_ changes from 2.5 m to 5 m, respectively. We also observe the moving conditions of nodes. The results are averaged over 10 simulated topologies.

**Figure 1:**
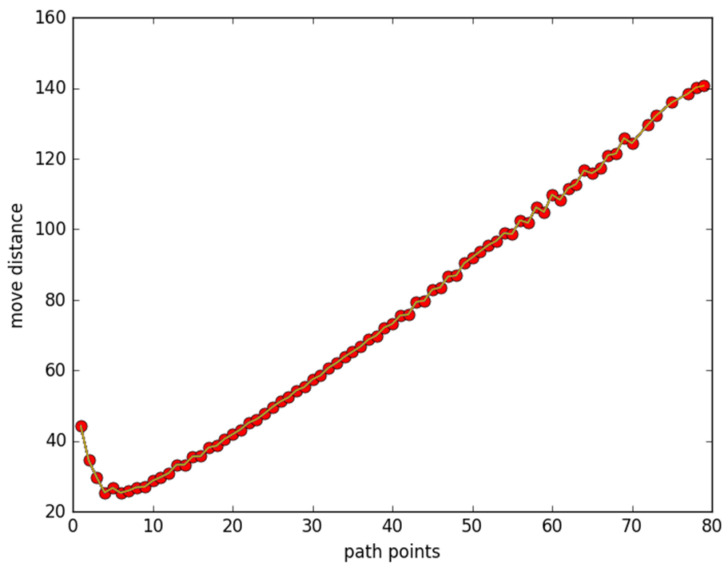
Moving distance changes with number of discrete points.

Figure 1 plots how the total moving distance changes as a function of the number of discrete points in the path, with 80 nodes located randomly in the area. Discrete points in the path are produced using the following function: *y*= 0.1(*x* − 20)(*x* − 10) (0 ≤ *x* ≤ 100). Other parameters are set as *d* = 0.5, *R*_1_ = 2.5, *R*_2_ = 5, and *β* = 0.5, respectively. As can be seen in Figure 1, there exists turning points in the curve on about five discrete points. At first, with few discrete points, all nodes have to move to their closest places. With the increase in the number of points, the moving distance decreases. However, as the number of points increased to some critical value (about eight here), more movements would be required to meet the covering expectations of all points.

**Figure 2:**
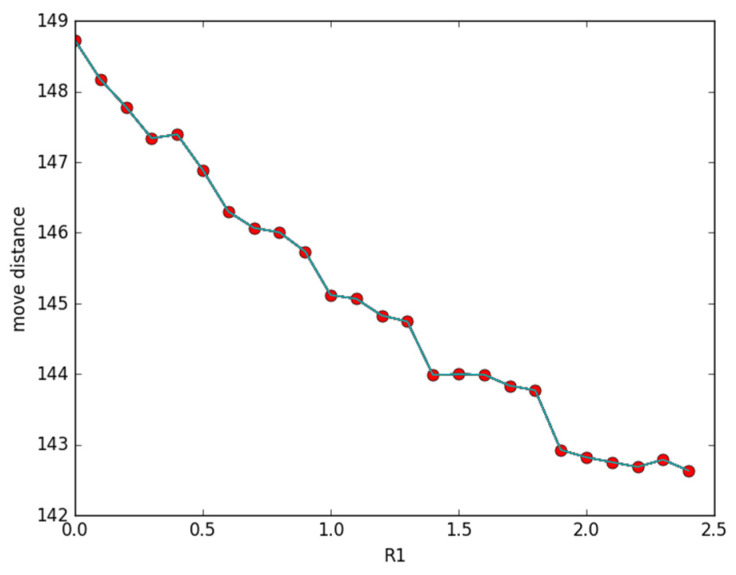
Moving distance changes with *R*_1_.

Figure 2 illustrates the changing conditions of the total moving distance according to variations in the sensing radius. We also use the same curve function, *y*, to generate 80 discrete points in the path, and then deploy 120 nodes randomly in the region. *R*_1_ ranges from 0 to 2.5 m, and the other parameters are set as *d* = 0.5, *β* = 0.5, and *R*_2_ = 5 m, respectively. It can be noticed from Figure 2 that moving distance decreases gradually in accordance with the increase in *R*_1_. This is because when *R*_1_ increases, the covering probability of all discrete points around it will become larger.

**Figure 3:**
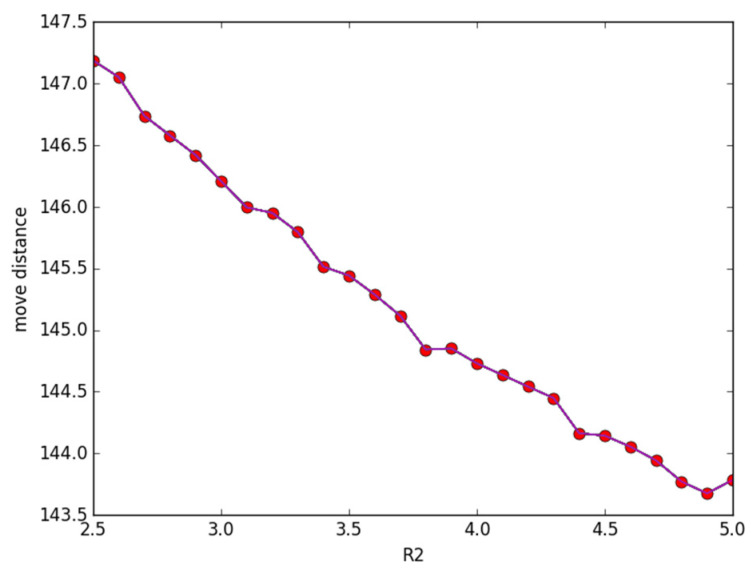
The change in *R*_2_ with the moving distance.

Figure 3 plots how the total moving distance changes according to *R*_2_. The parameters evaluating covering performance are set as *R*_1_ = 2.5, *d* = 0.5, and *β* = 0.5, respectively, while *R*_2_ changes from 2.5 m to 5 m. The other parameters are the same as in Figure 2. We observe that in Figure 3, with the increase of *R*_2_, the moving distance decreases. This phenomenon is caused by some discrete points being out of monitoring at the beginning while they are covered by nodes with the increase in *R*_2_.

**Figure 4:**
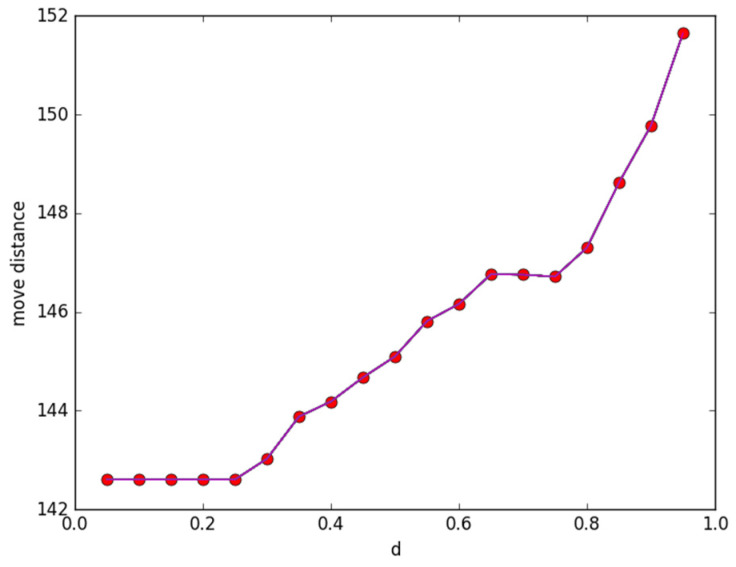
The change in *d* with the moving distance.

Figure 4 presents the changing condition of moving distance according to threshold *d*. In the initialized phase, we randomly produce 120 sensors in the monitoring area. Additionally, the same path function as in Figure 1 is taken to generate 80 discrete points. The covering probability threshold is ranged in (0.05, 0.95), and the other parameters are set as *β* = 0.5, *R*_1_ =2.5, and *R*_2_ = 5, respectively. We change the covering probability threshold *d* gradually. It can be observed from Figure 4 that, with the increase in *d*, the moving distance increases accordingly.

**Figure 5:**
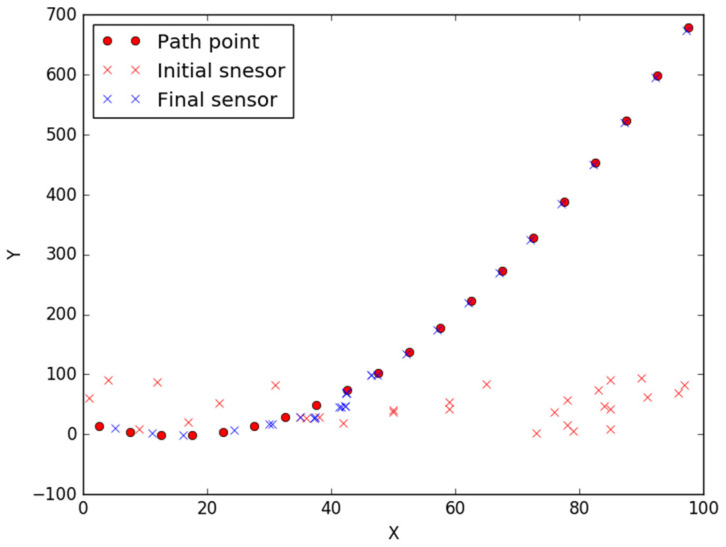
The movement of nodes.

Figure 5 shows the conditions of node deployment and movement in order to cover all discrete points in the path. Twenty discrete points are generated through the function of *y* = 0.1 × (*x* − 20) × (*x* − 10) (0 ≤ *x* ≤ 100), and then 30 nodes are located randomly in the area of 100 × 100 m^2^. The other parameters are set as *d* = 0.5, *β* = 0.5, *R*_1_ = 2.5, and *R*_2_ = 5, respectively. We observe from the curve that nodes move efficiently to save energy.

## 4. Largest Path Coverage Lifetime

### 4.1. Problem Analysis

#### 4.1.1. Marks

This section introduces the symbols that will be used in later aspects.

- E(*s*): The remaining battery level of node *s*, which is also called the lifetime of *s*;
- S(*v*): The collection of nodes that covered point *v*;
- C(*s*): The collection of points within the covering region of node *s*.

**Figure 6:**
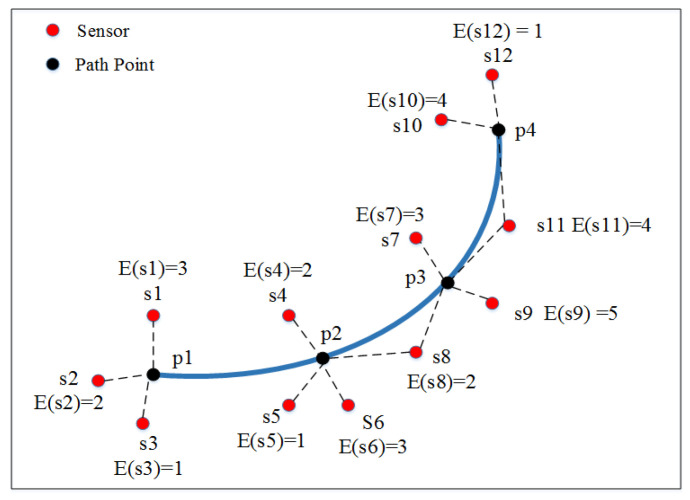
Path coverage in sensor network.

As illustrated in the network in Figure 6, twelve nodes and four points are located in the area. According to the definitions, here we have E(*s*_1_) = 3, S(*p*_1_) = {*s*_1_, *s*_2_, *s*_3_}, and C(*s*_8_) = {*p*_2_, *p*_3_}.

#### 4.1.2. Preliminaries

**Definition** **5.** *Coverage-* *weighted bipartite graph. In a sensor network, the graph is built as follows: B = (V*_1_*, V*_2_*, E). Here, the set V*_1_* is composed of vertices representing the corresponding nodes, and the collection V*_2_* contains vertices marking the corresponding points. When v belongs to collection C(u), the tuple (u, v) is treated as an edge in E. The value of (u, v) represents the residual battery level of node u.*

For nodes that are usually deployed densely, only parts of them need to be activated to cover all points in some fixed time; the others turn to sleep mode.

**Definition** **6.** *Path coverage lifetime. It is defined as the period of time that starts at all points being covered completely by nodes, and ends at any point where all the nodes cannot be covered.*

**Figure 7:**
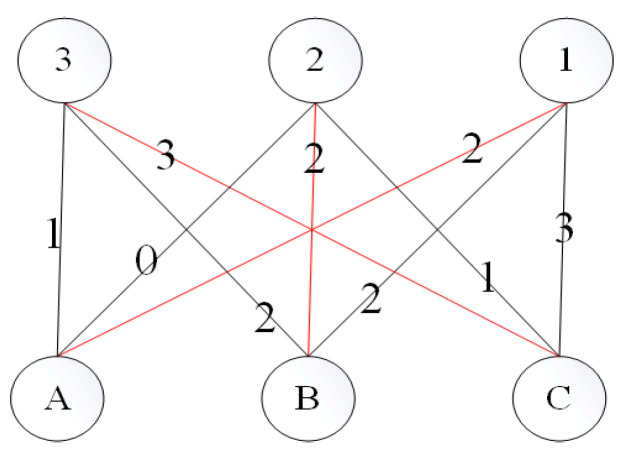
Largest weighted matching.

**Definition** **7.** *Largest Weighted bipartite Match (noted as LWM in later chapters). A match is defined in a graph G as a collection of edges that are vertex-disjoint. For a weighted bipartite graph, if the summation of values of all edges is the largest among all situations, then the weighted bipartite graph value is called LRM. As in Figure 7, A,B,C and 1,2,3 within each circle represents six different vertices, while the other numbers represent the value of each edge, respectively. Here LRM is {(1, A), (2, B), (3, C)}, with the summation of values even*.

#### 4.1.3. Problem Description

Considering the following problem: assuming multiple nodes are located randomly along a path, we first separate them into *h* partitions, then seek a way to schedule them in turn for path coverage with the largest monitoring lifetime (noted as PCLL). As in Figure 5, two partitions of nodes are formed: *T*_1_ = {*s*_1_, *s*_4_, *s*_7_, *s*_10_} and *T*_2_ = {*s*_2_, *s*_3_, *s*_5_, *s*_6_, *s*_8_, *s*_9_, *s*_11_, *s*_12_}, respectively. *T*_1_ is first used to monitor the path, and then *T*_2_ is taken to execute the task after some nodes in *T*_1_ exhaust their energy. *T*_1_ and *T*_2_ might not be the best partitioning solution; thus, some heuristic regulations are necessary for seeking a nearly optimal way.

In order to solve the PCLL problem, we decompose it into four steps: (1) separate the path into some discrete points, and divide all nodes into *h* partitions, respectively; (2) for the partitions that cannot cover all points completely, combine some of them to cover all points; (3) schedule all partitions to maximize the covering lifetime; and (4) schedule nodes within each partition to extensively maximize the covering lifetime. The mechanism for solving PCLL is formed through the combination of solutions of the above steps.

### 4.2. Largest Path Coverage Lifetime Algorithm

#### 4.2.1. Nodes Partitioning

Before the phase of node partitioning, we also use Algorithm 1 to make the path discrete, which is omitted here.

For a fixed path *P* and a collection of distributed nodes *S*, *h* partitions of nodes *T*_1_, *T*_2_, …, and *T_h_* are found, each of which can cover some points on path *P*. The collection S(*p*) of point *p* is separated into *k* partitions randomly, and each covers some number of points. The nodes partitioning solution is given in Algorithm 5. It loops *h* × *n* times since some points from each S(*p_j_*) are removed randomly in a round; thus, the algorithm consumes a total of O(*hnm*) energy.
**Algorithm 5:** Nodes-partition (*S*, *Q*)/* Input: deploy nodes collection *S* = {*v*_1_, *v*_2_, …, *v_n_*}, and points collection *Q* = {*p*_1_,…,*p_m_*} in *P*. Output: partitions collection of nodes. */ 1. Note S(*p_i_*) be the collection of nodes covering point *p_i_*; 2. Note *T*_1_, *T*_2_, …, *T_h_* be collection of node partitions; 3. *S*_1_ = Ø; 4. For (1 ≤ *i* ≤ *h*) & (1 ≤ *j* ≤ *m*) 5.     randomly pitch a subset *S*_0_ of S(*p_j_*); 6.     *T_i_* = *T_i_* + (*S*_0_ − *S*_1_); 7.     S(*p_j_*) = S(*p_j_*) − (*S′* − *S*_1_); 8.     *S*_1_ = *S*_1_ + (*S*_0_ − *S*_1_); 9. Return *T*_1_, *T*_2_, …, *T_h_*.

#### 4.2.2. Combine Partitions

After obtaining *h* partitions of nodes, a partition combination is needed to achieve the object that each new partition can cover all points completely. The problem is defined as follows: given *h* partitions of nodes *T*_1_, *T*_2_, …, *T_h_* and a collection of points *Q*, produce new collections of nodes *W*_1_, *W*_2_, …, *W_r_* (*r* ≤ *h*), making each collection *W_i_* cover all points in collection *Q*.

The solution is as follows: (1) first, arrange all partitions according to the number of points covered, and then take the first partition *T_i_* out of them; if *T_i_* can cover all the points, it is merged into collection *W_i_* and then removed from the partitions; (2) otherwise, we put *T_i_* into collection *W_i_* and then fetch the next partition *T_i+_*_1_. (3) In the case where *T_i+_*_1_ is able to cover the point left by *W_i_*, it is merged into *W_i_*, too; execute the above procedure continuously, stopping only when all points are covered by *W_i_* or all partitions are handled completely. (4) In the case that some points are left by *W_i_*, we check if there is a collection *W_j_* which is able to cover all points, and all remained partitions will be merged in a new collection *W_j_* with the combination of *W_i_*. In Algorithm 6, computing the number of points being covered by nodes takes O(*hnm*), sorting partitions takes O(*h*log*h*), and combining partitions takes O(*h*^2^). Thus, the algorithm totally consumes O(*hnm* + *h*^2^).
**Algorithm 6:** Combine-partition (*T*, *Q*)/* Input: collection *T* = {*T*_1_, *T*_2_,…, *T_h_*} of node partitions, collection *Q* = {*p*_1_,…,*p_m_*} of points in *P* Output: new partition collections *W*_1_, *W*_2_, …, *W_r_* (*r* ≤ *h*), each covers all points in collection *Q*. */ 1. Arrange *T* by quantity of covered points by *T_i_*, denoted by *T* = {*T*_1_, *T*_2_, …, *T_h_*}. *2. r* = 1; *T*_0_ = *T*; *S*_0_ = *Q*; 3. If (*r* ≤ *h*) 4.    Set *W_r_* be zero, *j* be one, respectively; 5. If (*T*_0_ is not null) & (*S*_0_ is not null) & (*j* ≤ *h*) & (|C(*T_j_*) − (*Q* − *S*_0_)| ≤ 0) 6.    *j*++; *W_r_* = *W_r_* + { *T_j_* }; *T*_0_ = *T*_0_ − *T_j_*; *S*_0_ = *S*_0_ − C(*T_j_*); *j*++; 7. If (*S*_0_ is null collection) 8.    *r*++; else if (*r* ≤ 1) 9. Return zero; else 10. Merge all collections in *T*_0_ with *W_r_* − 1; Merge all collections in *W_r_* with *W_r_* − 1; *r*--; 11. Return *W*_1_, …, *W_r_*.

#### 4.2.3. Partition Schedule

The partition schedule can be defined as follows: given some partitions of nodes, schedule them to achieve the largest lifetime of path coverage. It is obvious that the largest lifetime can be achieved by executing each partition once. The algorithm is given in Algorithm 7, which consumes time O(*r*).
**Algorithm 7:** Partition schedule (*W*)/* Input: partition collections *W* = {*W*_1_, *W*_2_, …, *W_r_*} (*r* ≤ *h*), each covers all points in *Q* = {*p*_1_,…,*p_m_*}. Output: the schedule of collections in *W* */ 1. Calculate expected lifetime for each *W_i_*; 2. For (1 ≤ *i* ≤ *r*) 3.    Command nodes in collection *W_i_* covering points in collection *P*; 4. Return.

#### 4.2.4. Intra-Schedule

Intra-scheduling aims to seek a method of making nodes in activating or sleeping mode, thus achieving the largest coverage time. To solve this, we first construct a coverage-weighted bipartite graph *G* and then seek an LRM in *G*. As a result, it is able to schedule nodes with the largest residual battery level. If they are not able to cover all points, we continue to construct the coverage-weighted bipartite graph *G_0_* for those points left over, which stops when all points are covered, or the remaining nodes cannot cover all points. The pseudo-code is described in Algorithm 8. As illustrated, finding the largest weighted matching consumes (*n*^2^*m*); thus, the total time consumed is O (*n*^2^*m*^2^*w*), where *w* is the largest value.
**Algorithm 8:** Intra-partition scheduling (*W_i_*, *Q*)/* Input: node collection *W_i_* covering all points in collection *Q* = {*p*_1_,..,*p_m_*}. Output: schedule of nodes */ 1. While nodes in *W_i_* cover all points in *Q* 2. Produce *G* = (*V*_1_,*V*_2_,*E*), where *V*_1_ denotes nodes set in *W_i_*, and *V*_2_ denotes points set in *Q*; 3. Find a LRM (noted as *M*) in *G*; 4. For each vertex without matching in *V*_2_ 5.    Denote unmatched vertices set by *V*_2′_ in *V*_2_; 6.    Induce a new sub-graph *G*_0_ from *V*_1_ and *V*_2′_; 7.    Find a LRM in *G*_0_; 8. For element *v* in *V*_2_, note M(*v*) as the element in collection *V*_1_ matching some element of *v* in collection *V*_2_, and the one in M(*v*) with least battery be *ver*_0_; 9. For element *v* in collection *V*_2_, schedule M(*v*) to cover point *v* and point M(*v*), thus each element in collection *V_i_* cost *ver*_0_ battery; 10.   Delete elements in *W_i_* out of service. 11. Return.

### 4.3. Simulations

We use simulations to further evaluate the effect of the number of nodes on the largest path coverage lifetime. As there is only one existing work analyzing path coverage in specialized situations, it is unfeasible to compare the simulation results with former works. A given path is divided into ten discrete points, and then a group of sensors is deployed around these points. The points covered by each sensor are also continuous. Due to space limitations, we only consider the impacts of the following parameters, quantity of nodes, initial battery level, and sensing radius. For parameter setting, the path is separated into ten points, while the energy of each node is set to a random number in the interval [5,10]. We assume that each node can cover one to three discrete points. The results are averaged over 10 simulated topologies.

**Figure 8:**
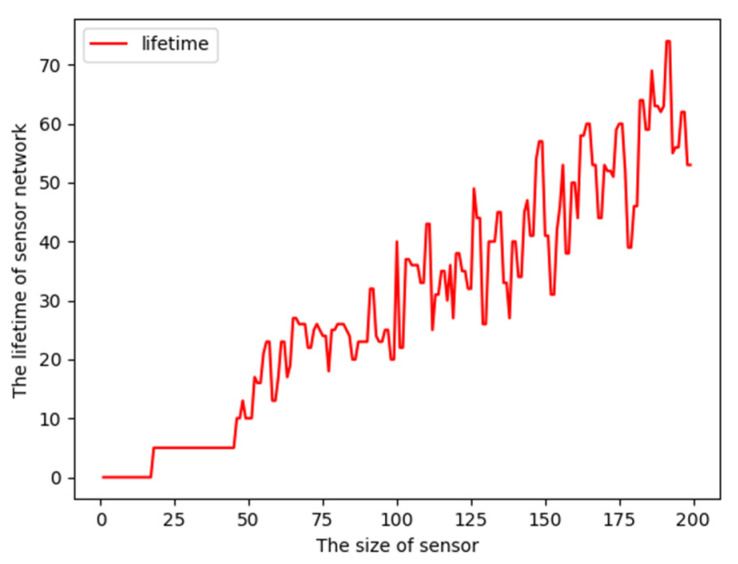
Lifetime changes with size of nodes.

The lifetime function curve changing with the size of nodes is illustrated in Figure 8. From Figure 8, we know that with a small number of nodes, the path coverage lifetime is zero. This is because of the lack of enough nodes to cover all points. With the number of nodes increasing, the coverage lifetime increases gradually as more nodes are engaged to cover points.

**Figure 9:**
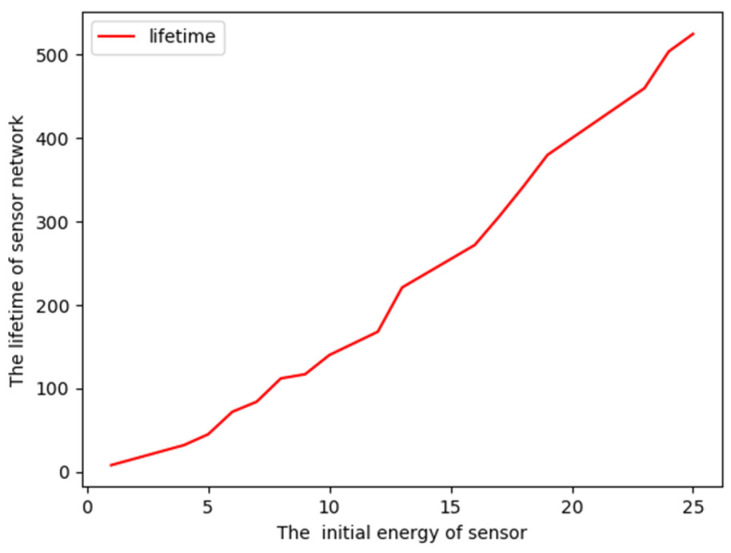
Lifetime changes with initial battery level.

The effect of the least initial battery level on the largest lifetime is given in Figure 9. Here, 200 nodes are located randomly around 10 discrete points in the path, and each node is equipped with the same battery initially. From Figure 9, we observe that the coverage lifetime also increases with the increase in the initial battery level of nodes, which enables nodes to monitor for a longer time.

**Figure 10:**
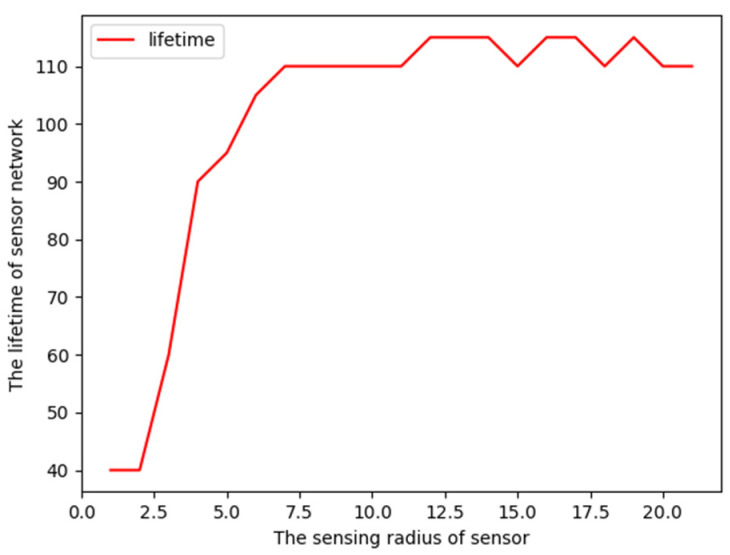
Lifetime changes with sensing radius.

The changing condition of coverage lifetime with sensing radius is also investigated in Figure 10. The path is separated into 10 points, around which are located 200 nodes randomly, and each node is equipped with the same energy valued randomly in the interval [5,10]. From Figure 10, we see that the coverage lifetime is positively related to the sensing radius, i.e., the larger the sensing radius, the bigger the coverage lifetime. However, after the sensing radius increases to a certain threshold, the coverage lifetime does not increase any longer. This is because, in the situation where the sensing radius is equal to the threshold, the nodes can cover the entire region completely.

## 5. Conclusions

In this paper, we have presented algorithms for two path coverage problems that had not been considered before: path coverage with the fewest movements of nodes, and node scheduling for maximizing path coverage lifetime. We first separated each problem into several sub-problems and then solved them one by one, and the original problem was finally solved through the combination of all sub-problems. For the first problem, the NP-hardness of the problem was proved, and the fewest movements were achieved through finding redundant nodes and paths; while for the second one, the largest bipartite matching was utilized to schedule partitions of nodes for monitoring. Moreover, curve disjunction was used on both algorithms to divide the path into points, which enables the proposed algorithm to be expanded to common sensor networks. We also analyzed the time complexities of the proposed schemes, and further evaluated the performance through experiments. However, the performance of the proposed algorithms was only evaluated under the experimental circumstance; we yet need to carry out some further work in actual sensor network-related scenarios to validate their effectiveness, e.g., multimedia sensor networks, health care sensor networks, traffic monitoring networks, etc. Moreover, how the optimality of each sub-step in the proposed algorithms can be proved also needs some further investigation. As for future work, we plan to seek results for the above-mentioned limitations of the work.

## Figures and Tables

**Table 1:** Comparison of the coverage algorithms.

Algorithms	Application Field	Object
MND [5]	point coverage	nodes movement
MTPCA [7]	point/region coverage	nodes movement
MSCP [8]	point/region coverage	nodes movement
MSPA [9]	barrier coverage	nodes movement
ABC [11]	point coverage	network lifetime
MC-MIP [13]	point coverage	network lifetime
MLCS [14]	point coverage	network lifetime
MSPA [16]	barrier coverage	network lifetime
HBOA [18]	point coverage	network lifetime
Our proposal	path coverage	nodes movement/network lifetime

## Data Availability

The experimental data was collected by the authors, and is not publicly available due to privacy.
